# Avicequinone C Isolated from *Avicennia marina* Exhibits 5α-Reductase-Type 1 Inhibitory Activity Using an Androgenic Alopecia Relevant Cell-Based Assay System

**DOI:** 10.3390/molecules19056809

**Published:** 2014-05-23

**Authors:** Ruchy Jain, Orawan Monthakantirat, Parkpoom Tengamnuay, Wanchai De-Eknamkul

**Affiliations:** 1 Department of Pharmaceutical Technology, Faculty of Pharmaceutical Sciences, Chulalongkorn University, Bangkok 10330, Thailand; E-Mails: ruchyj@gmail.com (R.J.); parkpoom.t@chula.ac.th (P.T.); 2 Research Unit of Natural Product Biotechnology, Faculty of Pharmaceutical Sciences, Chulalongkorn University, Bangkok 10330, Thailand; 3 Division of Pharmaceutical Chemistry, Faculty of Pharmaceutical Sciences, Khon Kaen University, Khon Kaen 40002, Thailand; E-Mail: oramon@kku.ac.th; 4 Department of Pharmacognosy and Pharmaceutical Botany, Faculty of Pharmaceutical Sciences, Chulalongkorn University, Bangkok 10330, Thailand

**Keywords:** avicequinone C, *Avicennia marina*, 5α-R1 inhibitory activity, cell-based bioassay

## Abstract

*Avicennia marina* (AM) exhibits various biological activities and has been traditionally used in Egypt to cure skin diseases. In this study, the methanolic heartwood extract of AM was evaluated for inhibitory activity against 5α-reductase (5α-R) [E.C.1.3.99.5], the enzyme responsible for the over-production of 5α-dihydrotestosterone (5α-DHT) causing androgenic alopecia (AGA). An AGA-relevant cell-based assay was developed using human hair dermal papilla cells (HHDPCs), the main regulator of hair growth and the only cells within the hair follicle that are the direct site of 5α-DHT action, combined with a non-radioactive thin layer chromatography (TLC) detection technique. The results revealed that AM is a potent 5α-R type 1 (5α-R1) inhibitor, reducing the 5α-DHT production by 52% at the final concentration of 10 µg/mL. Activity-guided fractionation has led to the identification of avicequinone C, a furanonaphthaquinone, as a 5α-R1 inhibitor with an IC_50_ of 9.94 ± 0.33 µg/mL or 38.8 ± 1.29 µM. This paper is the first to report anti-androgenic activity through 5α-R1 inhibition of AM and avicequinone C.

## 1. Introduction

*Avicenna marina* (AM), commonly called the grey or white mangrove, is a species of mangrove trees belonging to the Acanthaceae family. Traditionally it is used in Egypt to cure skin diseases [1] against fish stings, ringworms, sores, boils, skin ulcers and scabies [2]. It has also been used as a contraceptive [2] and in treating rheumatism [3]. In the literature, AM has been reported to exhibit antifertility [4], anticancer [5], antimicrobial [6] and antitumor [7] activities. Phytochemically, AM has been found to contain a variety of natural product groups, including naphthalene derivatives, flavones, iridoid glucosides, prenylpropanoid glycosides, abietane ditrerpenoid glucosides, flavonoid terpenoids and steroids [1].

AM has been used as a contraceptive due to its effects on the body’s endrocrine system [2]. The exact mechanism through which AM causes contraception is not yet understood, but most oral contraceptives affect the steroidogenesis pathway through either increasing or decreasing hormones or their related receptor levels or affecting the activities of the enzymes involved [8,9]. One of the enzymes present in the steroidogenesis pathway is 5α-reductase (5α-R), which converts testosterone (T) to 5α-DHT through the reduction of the Δ^4,5^ double bond [10]. Overproduction of 5α-DHT, a much more potent androgen, causes androgen-dependent diseases such as benign prostate cancer, acne and androgenic alopecia (AGA) [10]. Of these diseases, AGA is the main focus of this research work.

AGA is the major type of scalp hair loss in humans, affecting some 60%–70% of the worldwide population [11,12]. It affects 50% of males by the age of 50 and up to 70% of all males in their later life, while it affects only 25% of women by the age of 49% and 41% by the age of 69 years [13]. It is characterized by the miniaturization of the large, thick pigmented terminal hairs with diameters of greater than 0.03 mm into small, fine, non-pigmented vellus hairs with a diameter of 0.03 mm or less [11,14]. The miniaturization, due to the overproduction of 5α-DHT, results in the premature entry of the hair follicle into the catagen (transition) phase and the delay in the transition from the telogen (resting) to anagen (growth) phase, resulting in the shortening of the growth phase [15]. Therefore, one potential target for treating AGA is to inhibit this enzymatic reaction within the hair follicle. Two isoforms of this enzyme has been identified in different parts of the hair follicle, namely 5α-R1 and 5α-R type 2 (5α-R2) [10]. 5α-R1 is present in the dermal papilla cells, epidermal and follicular keratinocytes, while 5α-R2 is present in the inner layer of the outer root sheath, inner root sheath, interfollicular keratinocytes and might be present in the dermal papilla cells [16,17]. Different isoforms that catalyze the same reduction reaction are thus present in different parts of the hair follicle, however, only the dermal papilla cells are the site of action of 5α-DHT. In addition, they are the main regulator of hair growth as it plays an essential role in induction of new hair follicles and maintaining hair growth [18,19].

Therefore, in this study a cell-based assay system using dermal papilla cells commercially available as HHDPCs was used in order to evaluate the potential of AM as an 5α-R inhibitor, specifically for treating AGA. The assay system was coupled with a non-radioactive TLC detection technique. In addition, activity-guided fractionation through a preparative TLC technique was carried out in order to obtain pure bioactive compound(s) from the AM extract.

## 2. Results and Discussion

### 2.1. Expression of 5*α*-R1 in HHDPCs

**Figure 1:**
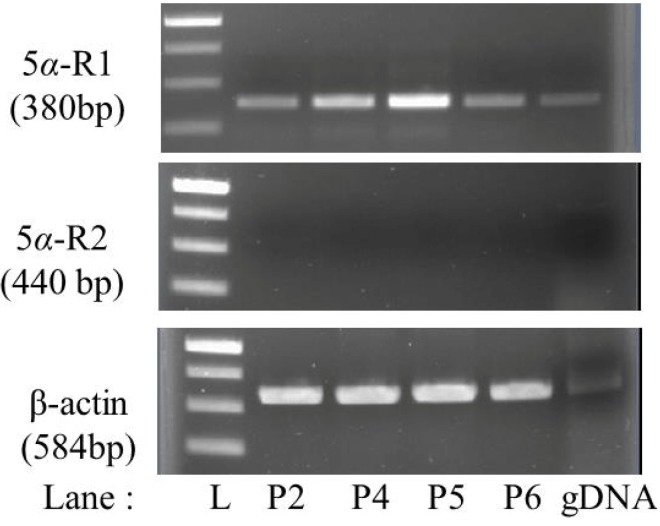
RT-PCR showing the expression of 5α-Rs and β-actin within HHDPCs. A 1% agarose gel showing, from the top, the expression of 5α-R1 (5α-reductase type 1, 380 bp), 5α-R2 (5α-reductase type 2, 440 bp) and β-actin (584 bp) within passages 2, 4, 5 and 6 of HHDPCs. The 1-kb DNA ladder (L) shows the band sizes of 1 kb and 750, 500 and 250 bp from the top down.

5α-R is responsible for the conversion of T into 5α-DHT causing AGA. Therefore, the presence of this enzyme within the HHDPCs was evaluated. The RT-PCR analysis revealed that the genes of 5α-R1 were expressed in passages 2, 4, 5 and 6 of HHDPCs, while 5α-R2 was not expressed in any of the passages (Figure 1). β-Actin, used as an internal control, was constantly expressed in all passages.

These results suggested that the type 1 enzyme, not type 2, is likely to have a direct role in hair growth regulation. This corresponds with previous works showing that the predominant form of 5α-R in HHDPC is 5α-R1 [10,16,17]. The two isoforms have 47% sequence similarity [10] and are known to be specifically distributed in different organs within the human body [10,16]. However, finding a number of reports in the literature that use 5α-R2 as the target enzyme for screening anti-AGA compounds is interesting [20,21,22]. This might be due to the previous clinical studies results showing that finasteride, a selective 5α-R2 inhibitor, decreases the 5α-DHT concentration and promotes hair growth in men with AGA [16,23]. This effect has been explained based on the results of immunohistochemical localization of 5α-R2 within the inner root sheath keratinocytes in the hair follicle rather than its direct effect on 5α-R1 within the HHDPCs which are known to be the main regulators of hair growth [10,16,17,24].

### 2.2. AM as 5*α*-R1 Inhibitor

Overproduction of 5α-DHT is caused by 5α-R, therefore one potential target to treat AGA is to inhibit this enzymatic reaction. A lot of research has been conducted using different cell types (e.g., transfected rat [25] or insect [21] cell lines) for a cell-based assay, or 5α-R enzymes isolated from unrelated organs (e.g., rat liver [26,27,28], prostate [29,30], epididymis [20] or human prostate [31]) for a cell-free assay. The use of these indirect cells or 5α-R enzymes has raised questions on the reliability of the available assays, therefore, we have developed a new cell-based assay using the appropriate target cells of the hair follicle, HHDPCs which are involved in hair growth and AGA.

The developed HHDPC-based assay system was used to evaluate the 5α-R1 inhibitory activity potential of AM, using dutasteride, a well-known potent inhibitor, as a positive control. The inhibitory activity was detected at 366 nm on the TLC plates based on the amount of 5α-DHT produced relative to the internal control (Cell+T), through a simple method of dipping the developed silica gel 60 F_254_ aluminum TLC plate into 42.5% phosphoric acid and then heating it at 120 °C for 20 min. As non-radiolabelled T was used as the substrate, the visual detection of 5α-DHT produced was recordable at 366nm using a TLC imager. There was no need to use the complicated detection techniques reported so far, which include radioactive image analyzers [27,30], TLC radioactive scanners [25], and HPLC radioactive detectors [21], or measuring the decrease in the radiolabelled T concentration using HPLC [20,28,29,31].

**Figure 2:**
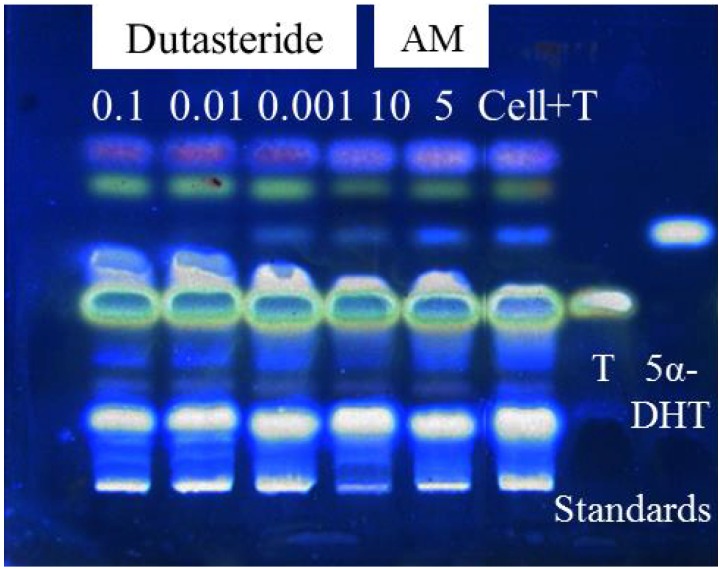
5α-R1 inhibitory activity of AM at 5 and 10 µg/mL (6.65% and 52% inhibition, respectively) and dutasteride at 1, 0.01 and 0.001 µg/mL (0%, 0% and 16.5% inhibition, respectively) using HHDPC-based assay system coupled with non-radioactive TLC detection technique. Cell+T is the internal control.

The results revealed that AM extract exhibited 5α-R1 inhibitory activity at the highest final non-toxic concentration of 10 µg/mL as it significantly (*p* < 0.05) reduced 5α-DHT production by 52% compared to a non-significant reduction of 6.65% at 5 µg/mL (Figure 2). Dutasteride exhibited complete inhibition at 0.1 and 0.01 µg/mL, while showing 16.5% reduction in 5α-DHT production at 0.001 µg/mL. The viability of the attached cells in the 96-well plate was 100.5% ± 2.02% (*n* = 3), confirming the non-toxic effect of the AM extract.

### 2.3. TLC Profile and Activity-Guided Fraction of AM

**Figure 3:**
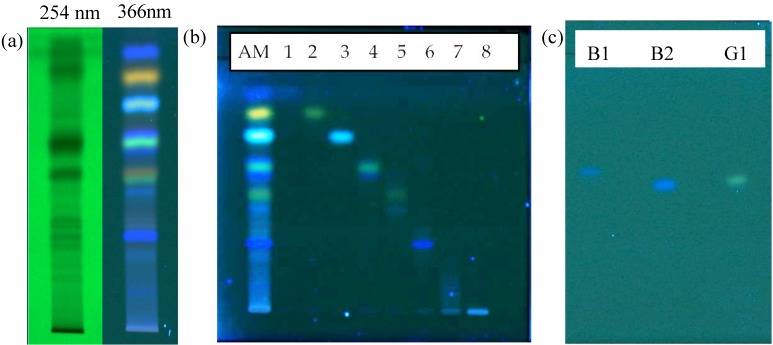
(**a**) TLC profile of the methanolic heartwood extract of AM visualized at 254 and 366 nm; (**b**) TLC plate visualized at 366 nm showing the isolated fractions; (**c**) Separation of B1, B2 and G1 from fraction “4” through double development of the TLC plate in 8:2 toluene–acetonitrile

**Figure 4:**
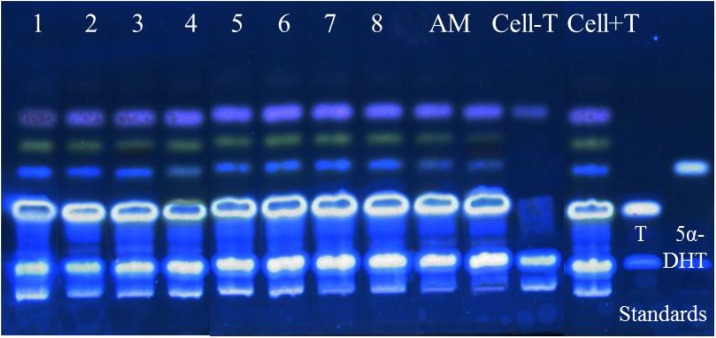
5α-R1 inhibitory activity assay of the eight AM fractions numbering 1–8 with 0, 19.2%, 13.3%, 50%, 18.9% 10.7%, 5.7% and 17.1% inhibition, respectively, comparing with the original AM extract (52% inhibition) using HHDPC-based assay system coupled with non-radioactive TLC detection technique. Cell+T is the internal control and Cell−T is the negative control.

The chemical complexity of AM extract was first observed by TLC. By using a silica gel plate and a mobile phase of toluene–acetonitrile–ethyl acetate–acetic acid in the ratio of 7:1:3:0.03, the constituents of AM appeared to be well separated as observed under the wavelengths of 254 and 366 nm (Figure 3a). Therefore, the preparative TLC technique was used to fractionate each observed band as fractions. Eight fractions were obtained from the preparative TLC, each of which showed clearly the presence of main constituent(s), especially at 366 nm (Figure 3b). Each fraction was then tested for 5α-R1 inhibitory activity at the final concentration of 10 µg/mL, and the results are shown in Figure 4. It can be clearly seen that fraction “4”, which is a mixture of at least three compounds, exhibited similar 5α-R1 inhibitory activity to that of the methanolic crude extract of AM. Therefore, further purification of this fraction was carried out using a double developing TLC system of toluene–acetonitrile in the ratio of 8:2 as the mobile phase, which lead to the isolation of two blue compounds and one green compound labeled as B1, B2 and G1 (Figure 3c).

**Figure 5:**
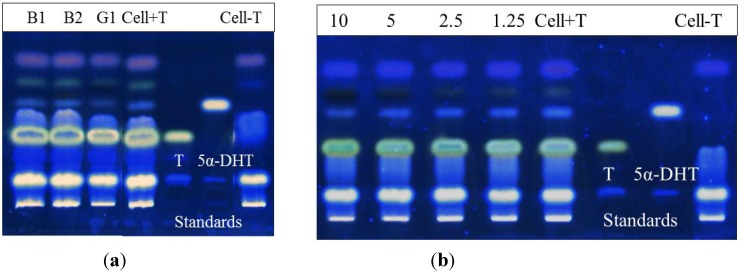
(**a**) 5α-R1 inhibitory activity of B1, B2 and G1 at the final concentration of 10 µg/mL; and (**b**) Dose-dependent response of G1 at 1.25, 2.5, 5 and 10 µg/mL (25.5%, 33.2%, 36.3% and 52% inhibition, respectively). Cell+T is the internal control and Cell−T is the negative control.

Each compound at the final concentration of 10 µg/mL was again tested for 5α-R1 inhibitory activity. As shown in Figure 5a, only G1 alone showed 5α-R1 inhibitory activity similar to that of AM with a 50% reduction in 5α-DHT production, giving an IC_50_ of 9.94 ± 0.33 µg/mL (Figure 5b) while B1 and B2 showed only 15% and 12% inhibitory activity, respectively.

**Figure 6:**
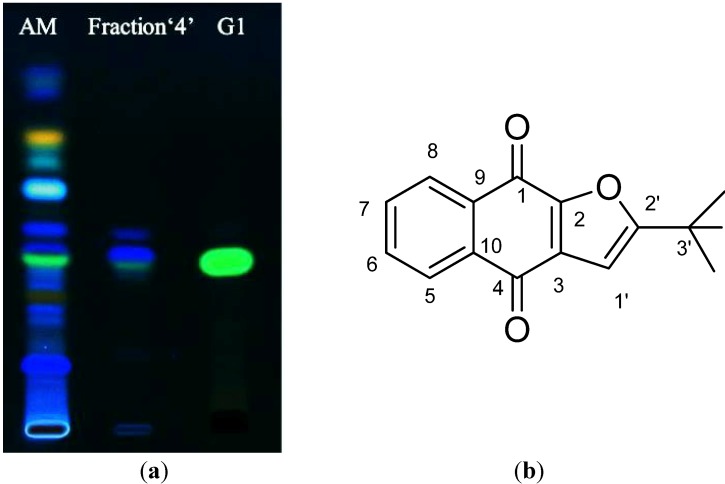
(**a**) Purity check of G1 compared with AM extract and fraction 4 by a silica gel TLC plate developed by toluene: acetonitrile in the ratio of 8:2 and visualized under 366 nm; (**b**) Structure of G1, identified as avicequinone C.

Subsequently, G1 obtained from the preparative TLC was further purified by semi-preparative HPLC. This was achieved through the application of 45 mg of G1 in 450 µL of DMSO to a TSK gel ODS (2 × 25 cm, 5 µm) column using 30% acetonitrile as a mobile phase with a flow rate of 9 mL/min. G1 was eluted as a major pure compound peak at a retention time of 78 min (Figure 6a).

NMR analysis of G1 was based on the spectral data of both ^1^H-NMR and ^13^C-NMR. By comparison with previously reported ^1^H-NMR and ^13^C-NMR data [32,33] (Table 1), G1 was identified as naphtho 2'-(1-hydroxy-1-methylethyl)[2,3-β]furan-1,4-dione, or avicequinone C (Figure 6b) with a molecular formula of C_15_H_12_O_4_ and a molecular weight of 256.

**Table 1:** NMR spectra data of G1 and avicequinone C in CDCl_3_.

Position	G1		Avicequinone C [32,33]	
	^1^H(mult., *J* in Hz)	^13^C	^1^H(mult., *J* in Hz)	^13^C
1	-	173.4	-	173.3
2	-	151.8	-	151.6
3	-	131.3	-	131.2
4	-	180.8	-	180.7
5	8.16 (m)	126.9	8.14 (m)	126.8
6	7.75 (m)	133.9	7.73 (m)	133.9
7	7.75 (m)	133.7	7.73 (m)	133.7
8	8.21 (m)	126.8	8.18 (m)	126.8
9	-	132.5	-	132.4
10	-	133.1	-	
1'	6.82 (s)	102.6	6.80 (s)	102.6
2'	-	167.9	-	168.1
3'	-	69.4	-	69.3
4'	1.69 (s)	28.8	1.67 (s)	28.7
5'	1.69 (s)	28.8	1.67 (s)	28.7

Avicequinone C has a furanonaphthaquinone structure. It was first isolated from AM in 2000 by Ito. *et al*. [32]. The compound has been shown to have antimicrobial and antiproliferative activities [34]. In this study, avicequinone C showed its 5α-R1 inhibitory activity, with an IC_50_ value of 38.8 ± 1.29 µM. Although this value indicates moderate potency, it is still better than or equivalent to the reported IC_50_ values of many natural products isolated from plants, for example, the IC_50_ values of 112 µM for soyasaponin1 from *Pueraria thomsonii* [20], 85 µM for artocarpin from *Artocarpus incises* [35], 40 µM for emodin from *Polygonum multiflorum* Thunb [36], 31.7 µM for triolin from *Torillis japonica* [37], 390 µM, 230 µM, 220 µM and 220 µM for 1,7-diphenylhept-4-en-3-one, dihydroyashabushiketol, 5-hydroxy-7-(4''-hydroxy-3''-methoxyphenyl)-1-phenyl-3-heptanone and 5-hydroxy-7-(4''-hydroxyphenyl)-1-phenyl-3-heptanone from *Alpinia officinarum* [38] and 44 µM, 103 µM and 48 µM for (‒)-cubebin, (‒)-3,4-dimethoxy-3,4-desmethylenedioxycubebin and piperine from *Piper nigrum*, respectively [39].

The presence of the 1,4-naphthoquinone nucleus of avicequinone C might be important for the activity, as it is in alizarin, which exhibited 5α-R1 inhibitory activity in both cell-free and cell-based assays with IC_50_ values of 3 and 6 µM, respectively [25]. Purpurin, an anthraquinone, also exhibited 5α-R1 inhibitory activity in a cell-free assay with an IC_50_ of 2 µM [25]. However, to date only these two naphthoquinones have been identified as 5α-R inhibitors and no structure-activity relationships have been conducted on this group of compounds.

## 3. Experimental

### 3.1. Chemicals, Enzymes and Reagents

All of the organic solvents used were analytical grade and purchased from RCI Labscan (Bangkok, Thailand). Ultrapure grade dimethyl sulfoxide (DMSO) was purchased from Ameresco^®^ (Framingham, MA, USA). T and 5α-DHT were purchased from Sigma-Aldrich (St. Louis, MO, USA). Dutasteride was purchased from BDG Synthesis (Wellington, New Zealand). Agarose-LE was purchased from Affymetrix (Santa Clara, CA, USA). Mesenchymal stem cell medium and its supplements were purchased from Sciencell Research Laboratories (Carlsbad, CA, USA). Fetal bovine serum, 100× antibiotic-antimycotic solution, 10× PrestoBlue^®^ (Life Technologies, Carlsbad, CA, USA), RPMI medium, 50× Tris-acetate-EDTA (TAE) buffer, 0.25% trypsin-EDTA and Platinum^®^ *Taq* polymerase kit were purchased from Invitrogen (Grand Island, New York, NY, USA). A GeneRuler 1-kb DNA ladder was purchased from Thermo Fisher Scientific (Pittsburgh, PA, USA). RNeasy^®^ mini kit was purchased from Qiagen (Valencia, CA, USA). DNase I enzyme, EDTA, first-strand cDNA synthesis kit, dATP, dTTP, dCTP and dGTP were purchased from Fermentas (Waltham, MA, USA).

### 3.2. Plant Material and Extraction

The heartwood of AM was obtained from a local Thai-Chinese medicinal store and was first ground into powder and further subjected to maceration using 100% methanol at room temperature for two days. The methanolic extract was then evaporated to dryness at 45 °C using a rotary evaporator (Rotavapor R-210, Buchi, Flawil, Switzerland) and kept at −20 °C until used.

### 3.3. Culturing of HHDPCs

HHDPCs, obtained from Sciencell Research Laboratories, were grown in mesenchymal stem cell medium containing 5% fetal bovine serum (FBS), mesenchymal stem cell medium supplement, and 1× antibiotic-antimycotic solution at 37 °C in 5% CO_2_. The cells between passages 2 to 6 were used in this study.

### 3.4. Checking for the Presence of 5*α*-R in the HHDPCs

Reverse-transcriptase polymerase chain reaction (RT-PCR) was used to identify the isoforms of 5α-R (*i.e.*, 5α-R1 and/or 5α-R2), expressed in HHDPCs from passages 2, 4, 5 and 6. The forward and reverse primers for the two isoforms of 5α-R and β-actin, shown in the Table 2, were designed from the protein region of the full length sequence obtain from the NCBI GenBank using Clone Manager (Scientific & Educational Software, Cary, NC, USA) and made to order at 1st Base Laboratories (Selangor, Malaysia). The PCR products were analyzed using 1% agarose gel electrophoresis.

**Table 2:** Forward and reverse primers and expected sizes of 5α-Rs and β-actin.

Name	Primer pair 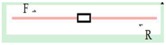	Expected size (bp)
5α-reductase type 1 (5α-R1) GenBank: NM_001047.2	F:5' ACTGCATCCTCCTGGCCATGTTC 3' R:5' GGCATAGCCACACCACTCCATGA 3'	380
5α-reductase type 2 (5α-R2) GenBank: NM_000348.3	F:5' AAGCACACGGAGAGCCTGAA 3' R:5' GCCACCTTGTGGAATCCTGTAGC 3'	450
β-actin (internal control) GenBank: NM_001101.3	F:5' ATGATGATATCGCCGCGCTC 3' R:5' GCGCTCGGTGAGGATCTTCA 3'	584

### 3.5. Cytotoxicity of AM on HHDPC

In order to obtain a suitable starting concentration for testing the inhibitory activity, the highest non-toxic concentration of AM was determined. HHDPCs were seeded at a cell density of 1 × 10^5^ cells/mL onto 96-well plates (100 µL of 10,000 cells/well). After 24 h, the cells were separately treated with 100 µL of AM or 1% DMSO (control). The concentration of AM ranges from 40, 20, 10 and 5 µg/mL with the final concentrations of 20, 10, 5 and 2.5 µg/mL, respectively. Cell viability was measured 24 h after the treatment using 1× PrestoBlue^®^ (Life Technologies) reagent in RPMI medium. In the presence of viable cells, PrestoBlue^®^ changes from a non-fluorescent blue color to a fluorescent purple-pink color, which is detected using the Multimode Detector DTX 880 (Beckman Coulter^®^, Indianapolis, IN, USA), a bottom-read fluorospectrophotometer with an excitation/emission of 535/615 nm. The results showed that AM was not toxic to HHDPCs, (*i.e.*, cell viability more than 85%) up to the final concentration of 10 µg/mL of AM. Therefore, this concentration was used as the starting concentration for the 5α-R1 inhibitory activity test.

3.6. 5*α*-R inhibitory Activity Test

HHDPCs were seeded at a cell density of 1 × 10^5^ cells/mL onto 96-well plates (100 µL of 10,000 cells/well). After 24 h, the cells were separately treated with 50 µL of 4 × 10^−4^ M T and 50 µL of 2% DMSO (internal control); 50 µL of 4 × 10^−4^ M T and 50 µL of 40 µg/mL AM; and 100 µL of 2% DMSO (negative control). The cells were treated for 48 h, before the cell culture medium was collected in Eppendorf tubes, and the attached cells were tested for cell viability using the 1× PrestoBlue^®^ (Life Technologies) reagent in RPMI medium in order to avoid false positive result. T and its product, 5α-DHT, were extracted from the cell culture medium using an equal volume of ethyl acetate. The ethyl acetate fraction was then dried, reconstituted with 20 µL of methanol and spotted on a TLC Silica gel 60 F_254_ aluminum plate (Merck, Darmstadt, Germany). The TLC plate was developed using toluene–acetone at a ratio of 8:2 as the mobile phase [30]. The developed TLC plate was dipped in a solution of 42.5% phosphoric acid and heated at 120 °C for 20 min, for the visual detection of 5α-DHT at 366 nm using a TLC reprostar imager (Camag, Muttenz, Switzerland), and the amount was quantified using an image analyzing program, Quantity One (Bio-Rad, Hercules, CA, USA). The inhibitory activity was determined through the decrease in 5α-DHT production relative to the internal control.

### 3.7. TLC Profile of AM

TLC was used to observe the complexity of the methanolic heartwood extract of AM. The TLC silica gel 60 F_254_ aluminum plate was spotted with 10 µL of AM at the concentration of 3.5 mg/mL and was developed in toluene–acetonitrile–ethyl acetate–acetic acid in the ratio of 7:1:3:0.03 as the mobile phase. The developed plate was then visualized under the wavelengths of 254 and 366 nm.

### 3.8. Isolation and Structural Analysis of Bioactive Compound(s) within AM

Preparative TLC was used to separate and isolate the compounds in AM. The extract was developed, using the same system as mentioned above, on a preparative TLC Silica gel 60 F_254_ glass plate and each band/fraction was isolated through scraping. The bands/fractions were then tested for 5α-R inhibitory activity using the developed assay system. The purity of active compound(s) were checked using HPLC before structure elucidations were conducted using ^1^H-NMR and ^13^C-NMR: ^1^H-NMR (400 MHz, CDCl_3_) *δ*_H_ (mult (*J* in Hz); H): 8.16 (*m*; 1H,H-5), 8.21 (*m*; 1H, H-8), 7.75 (*m*; 2H, H-6, H-7), 6.82 (*s*; 1H, H-1'), 1.69 (*s*; 2H, H-4', H-5'); ^13^C-NMR (100 MHz, CDCl_3_) *δ*c: 173.4 (C-1), 151.8 (C-2), 131.3 (C-3), 180.8 (C-4), 126.9 (C-5), 133.9 (C-6), 133.7 (C-7), 126.8 (C-8), 132.5 (C-9), 133.1 (C-10), 102.6 (C-1'), 167.9 (C-2'), 69.4 (C-3'), 28.8 (C-4', C-5').

### 3.9. Statistical Analysis

All of the experiments were performed in triplicate, and the data are presented as the means ± SD. One-way single factor ANOVA was used and a *p*-value < 0.05 was taken to be statistically significant.

## 4. Conclusions

A natural furanonaphthaquinone exhibiting 5α-R1 inhibitory activity (IC_50_ of 38.8 ± 1.29 µM) and identified as avicequinone C, a known compound, was successfully isolated from the heartwood of AM. This was accomplished by a simple activity-guided fractionation using TLC as a tool for both the cell-based assay detection and compound isolation. In addition, HHDPCs was used in this cell-based assay due to its properties of being the main regulator of hair growth as they are the only cells within the hair follicle that are the direct site of 5α-DHT action, and therefore, considered to be the right target and used for the first time to screen for anti-AGA compounds. Further studies on the modification of avicequinone C might lead to more potent 5α-R1 inhibitors with higher potential in treating AGA.
